# How does human capital affect food security? A perspective of specialization

**DOI:** 10.3389/fpubh.2025.1633830

**Published:** 2025-11-06

**Authors:** Huishuang Jin, Hui Liu

**Affiliations:** School of Economics, Hunan Agricultural University, Changsha, China

**Keywords:** specialization, human capital, mechanization, land, social, China

## Abstract

The world is facing a food crisis and the goal of zero hunger is facing increased uncertainty. Promoting the transition from traditional to modern agriculture is important for improving agricultural productivity. Human capital is the central factor in driving the transformation of agricultural modernization. Agricultural specialization, as an important symptom of agricultural modernization, is closely linked to the human capital of the rural workforce. And, previous studies on food security have focused mainly on the area of food crops planted. Changes in the cropping structure within food crops have been neglected, and exploration from the perspective of specialized operations can fill this gap. And, few studies have integrated human capital levels with the development of agricultural social services. Therefore, the study uses large-scale micro-survey data representative of China, adopts the HHI to measure agricultural specialization, and empirically analyzes the impact of human capital on agricultural specialization and the role played by the level of agricultural socialization services in this process based on models such as OLS. Studies have shown that the human capital level of Chinese farmers is low, and there is still much room for improvement in the degree of agricultural specialization. The regression results show that human capital contributes significantly to agricultural specialization. The mechanism test show that human capital can promote the specialization by enhancing the agricultural social services. The impact of human capital on specialized operations varies significantly across regions, business scales, land transfer practices, and social network conditions. Therefore, we need to strengthen investment in the level of rural human capital and accelerate the training of new professional farmers. Agricultural infrastructure should be increased and the agricultural service supply system should be improved. Effectively strengthening the coverage of agricultural services and improving the ability of agricultural socialized service organizations to link up with and lead farmers. In addition, land transfers and moderate-scale operations should be promoted, thereby accelerating the process of agricultural modernization.

## 1 Introduction

Agriculture is the foundation of the national economy, and a strong country is only as strong as its agriculture. As a big agricultural country, China has a wide variety of agricultural products and widespread diversification of production by farm households, with the vast majority of provinces (cities and districts) growing all of the major crops included in the statistics (especially rice, wheat, corn, soybeans, potatoes, etc.). Diversification opens up opportunities for farmers to cope with uncertain shocks such as markets and climate change (1), but it also leads to inefficiencies in the productivity of agricultural labor and makes it difficult to endogenize large-scale operations. Throughout the developed countries of global agriculture, division of labor and specialization have been key to improving efficiency. As early as last century, the United States has achieved the specialization of agricultural production, no state grows all of the seven major crops included in the United States statistics, and the number of farms operating a product reaches more than 90% of the farm population, which alone reduces the cost of production by 50 to 80%, and increases the value of agricultural products by about 40%. Promoting agricultural specialization has become an inevitable trend in China’s future agricultural development (2). However, the overall level of human capital in rural China is low at present, and aging and off-farming are prominent (3), so it’s urgent to improve rural human capital.

In 1974, the FAO first proposed that food security means ensuring that everyone has access to food for survival and health at all times. Since then, there have been several revisions. There is no consensus in academia on how to measure food security, and some scholars have used a weighted population method to measure food security in the United States (4). Some scholars have also based their assessments on grain supply capacity, using metrics such as grain planting area and grain production (5, 6). From the perspective of China’s agricultural situation, focusing on efficiency and encouraging farmers to specialize in their operations is an important way to promote the modernization of Chinese agriculture and ensure national food security (2).

From the existing research results, it is clear that capital accumulation is crucial for rural development (7), and investing in human capital for farmers is an important pathway to transform traditional agriculture (8). In particular, farmers’ human capital has multiple effects, not only effectively improving the source of household income, but also promoting the non-farm transfer of labor by investing in human capital such as out-of-home employment experience, upgrading of education, and advancement of technical skills (9, 10). In terms of out-of-home experiences, some scholars consider that off-farm employment will cause the diversification of farm households’ sources of income and the “Non-Grain Production” of cultivation, which will push farmers to invest more in capital-intensive crops (11) and shift to the cultivation of higher-yielding cash crops (12). However, it has also been pointed out that the rising opportunity cost of labor brought about by the transfer of agricultural labor has prompted farmers to plant more mechanized crops and thus increase productivity (13). Based on food security motives, households with non-farm employment do not invest in agricultural technologies that increase productivity and save time on the farm. They usually have two possible options to cope with the debt and income instability associated with migration. One is to withdraw from agricultural production activities and the other is to expand the area under food crops (14, 15). That is, non-farm employment enhances the food security of farm households (16), promotes agricultural specialization (17), and thus increases the efficiency of agricultural production (18). In terms of education, higher education is a strategy to mitigate food insecurity (19), the level of education of household members has boost the crop production (11), while the accumulation of financial knowledge reduces the area of grain cultivation (20). Age is another important factor affecting agricultural production.

Young, well-educated heads of households benefit the farm (11). Rural population aging drives capital factor deepening through agricultural factor restructuring (21), prompting farmers to reduce the proportion of cash crops grown and grow more food. The augmentation of agricultural mechanization level will promote a further increase in the share of grain cultivation (22). In addition, agricultural production conditions are another factor influencing the cropping structure (23), and road accessibility is conducive to agricultural production specialization (24).

Ensuring food self-sufficiency is especially critical in a country like China that places a high priority on food security, with more than 1.4 billion mu of arable land in need of food cultivation. In particular, in recent years China has been increasing its efforts to build high-standard farmland. High-standard farmland is prioritized for the production of rice, wheat, corn and other major grains. At the same time, food crops are much more mechanized than cash crops. As a result, most farmers are also specializing more toward specialization in food production. For example, the northeastern region of China is the ballast of food production, and the concept of “building a national food security industrial belt,” introduced in 2020, has given a strong impetus to specialized food production in the northeastern region.

In summary, most of the existing empirical studies on the structure of agricultural business focus on a single human capital factor, such as off-farm employment, education, age, etc., with a view to “grainification” or “non-grainification”. There is little literature dealing with agricultural specialization, and insufficient attention to restructuring within food or cash crops (such as reducing maize cultivation and increasing rice cultivation). Meanwhile, a stockpile of each type of human capital factor varies considerably within the farm household, and the use of a single factor to proxy for a farmer’s level of human capital may overestimate or underestimate its impact on agricultural production. In addition, mechanization is an important factor in the transformation and upgrading of agriculture, and service outsourcing is a main way for smallholder farmers to enjoy mechanization. It’s necessary to explore the influence of the current human capital level and agricultural mechanization level of Chinese farmers on agricultural transformation and upgrading.

Overall, existing studies have conducted extensive and useful explorations from different perspectives, but there is still some room for expansion and optimization, mainly in the following: (1) Existing research on food security focuses mostly on “grainification” or “non-grainification,” with insufficient attention paid to structural adjustment within food crops, and little literature on agricultural specialization. (2) Fewer studies have analyzed this process from the perspective of human capital, and the inner mechanisms of this process have not yet been fully clarified. In this regard, the paper will be improved as follows: first, the reality of agricultural production will be explored based on the perspective of specialization. This expands the research perspective; Second, considering the comprehensive nature of human capital and the reality that the degree of food mechanization in China is much higher than that of cash crops, a theoretical analysis framework is constructed to explore the channels of its role from the perspective of agricultural mechanization, which enriches the existing theoretical system; Third, relying on representative data with a Chinese dimension, we explore whether there is a heterogeneous effect of human capital on agricultural specialization. This provides a reference for promoting food security in regions in similar contexts.

The structure of this paper is as follows. Section 2 reveals the mechanism of human capital’s influence on agricultural specialization. Section 3 includes three parts: data source, variable selection, and model setting. Section 4 explains and discusses the results. Section 5 summarizes the main conclusions and gives policy recommendations.

## 2 Theoretical analysis framework

### 2.1 The direct impact of human capital on agricultural specialization

Human capital is the most important of all resources, expressed as the sum of stocks of knowledge, labor, health, etc. that generate economic value through labor (25), and directly affects the structure of agricultural operations mainly through internal and spillover effects. First, internal effects refer to the fact that human capital enhances its productivity by accumulating knowledge and skills (26). From Schultz’s theory of human capital, it is clear that population migration is an investment behavior that depends on the difference between the costs and benefits of migration. When the human capital stock of farming households is low, they are difficult to migrate for non-farm income and are unable to master new agricultural technologies to improve labor efficiency (27). At this time, physiological needs dominate, and farmers diversify their planting structure to achieve self-sufficiency. As the level of human capital accumulates, highly educated and skilled groups who migrate are more likely to earn a return on their investment in human capital, and their opportunity cost of agricultural labor increases, the more likely they are to decide to migrate for higher off-farm incomes, which in turn leads to farmers planting field crops that are less labor-demanding (28). In addition to influencing their own business decisions, the human capital of farmers has an impact on other farmers, which can also be said to generate spillover effects. In particular, “geographic” and “blood” ties in rural China have a strong bonding effect. Farmers’ own increased level of human capital, such as knowledge, skills and experience, may unconsciously transfer expertise to neighboring groups through communication and cooperation. This can lead to knowledge spillovers and skill diffusion, which in turn can help other farmers to improve their production methods (29). Higher levels of human capital imply a stronger profit motive, and they are more capable of absorbing and applying new knowledge and technology (30, 31). For the purpose of reducing the cost of information collection and agricultural procurement, their cooperation and communication with agricultural service organizations are more frequent, and knowledge is overflowing from specialized organizations to production and business farmers. At the same time, farmers tend to actively learn to imitate professional organizations, and are more likely to expand the scale of operation of a particular crop, aiming to achieve economies of scale and strengthen the level of agricultural specialization. Human capital contributes significantly to agricultural specialization.

### 2.2 The mechanism of agricultural socialization services

**Figure 1:**
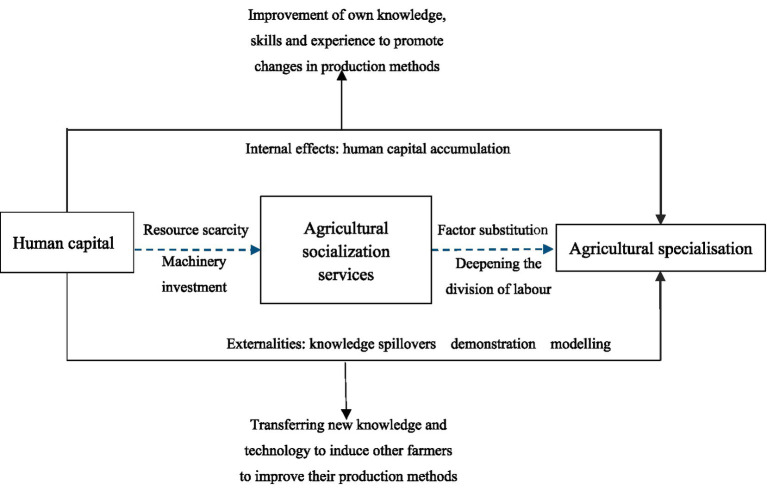
Analytical framework.

As stated by the theory of induced technological change, relative changes in factor prices due to changes in resource scarcity induce technological change and production adjustment (32). Investment in machinery, the largest investment in Chinese agricultural production, sets the stage for a deepening of the division of labor in agriculture (33). The deepening of the division of labor is a process of continuous exclusion of labor and can change the pattern of agricultural production and business through labor substitution effects. That is to say, the level of labor scarcity and price increases cause substitution between input factors, promoting farmers to convert the intensity of capital and labor inputs and adjust the structure of agricultural production (34). However, the sunk costs of purchasing a machine for self-employment are high, from this came the agricultural machinery service. Agricultural mechanization services reduce transaction costs by relying on social relations among rural acquaintances, compensate for the dilemma of labor shortage and aging, and improve the conditions of production, enabling producer to reduce the acreage of labor-intensive crops (35). First of all, farmers with high levels of human capital are more rational in their perception of new things, have a higher willingness to accept new technologies that improve the efficiency of production, and are more inclined to use agricultural machinery to replace labor (3), which enhances the capacity of the market for agricultural machinery services, and induces rational farmers to cultivate crops with a higher rate of labor, thus forming a horizontally concentrated and contiguous pattern of production. Secondly, farm equipment has a strong asset specificity, and it is easier for service organizations to provide standardized services in large-scale continuous crop areas and to maximize returns in the shortest possible time. In other words, the main body of agricultural machinery service will actively induce the scale cultivation of the same kind of crops in the main body of agricultural management (36), to promote the specialized management of agriculture. Thus, human capital contributes to agricultural specialization by influencing the development of agricultural social services (Figure 1).

## 3 Data and methodology

### 3.1 Study region and data sources

We use data from the Chinese Family Database (CFD)^1^, which began in 2011 with more complete information on Chinese rural households and Chinese grassroots units (village committees). Currently, the database has been updated to 2017 and covers a sample of 24,764 rural households and 15,247 urban households in 29 provinces (cities and districts) of China, with representativeness at the national, provincial urban and rural levels. Considering that only the 2017 questionnaire in the CFD database has more detailed statistics on Chinese agricultural production data. Therefore, we use 2017 data for empirical evidence. After cleaning up for missing values and outliers, a total of 6,275 valid farm household sample data from 476 villages were retained in 2017. The survey sample can better reflect the basic situation of human capital and agricultural specialization of farm households at the national level.

### 3.2 Agricultural specialization measurement

The use of specialization in agricultural production is to reduce the number of types of crops planted to maximize the concentration of resources and factors of production to the advantageous agricultural products, which is specifically manifested in the continuous planting produced by the horizontal division of labor (37), this can be measured using the Herfindahl–Hirschman Index (HHI) (38). The HHI is between 0 and 1, with higher values indicating a higher degree of planting specialization. When the HHI is equal to 1, it indicates that farmers specialize in the production and operation of a particular agricultural product; when the HHI is equal to 0, it indicates that farmers have completely diversified their operations. Combining the characteristics of CFD data, we construct the agricultural specialization index (Equation 1), which is measured by the formula:


$$\mathrm{HHI}=\sum_{j=1}^{n}S_{\mathrm{ij}}^{2}=\sum_{j=1}^{n}{\left(\frac{x_{\mathrm{ij}}}{x_{i}}\right)}^{2}$$ (1)

Where *S*_ij_ denotes the proportion of sown area *x*_ij_ of farmer i’s jth crop to his total sown area *x*_i_.

### 3.3 Human capital index measurement

The level of human capital in farm households usually has both quantitative and qualitative aspects (39, 40). To investigate the level of human capital more comprehensively, based on existing studies (29, 35, 41, 42), combining the characteristics of the samples, the human capital value of farming households is portrayed in terms of the number of household agricultural laborers, the highest level of educational attainment, the average health status of the population aged 16 years or older, and life cycles. At the same time, to avoid the subjectivity of artificial assignment, the weights of the indicators are objectively assigned through the entropy value method to roundly estimate the level of human capital (43). Among them, the life cycle indicator takes into account the age structure of Chinese rural family members and the characteristics of agricultural production, and divides the life cycle of farming households into four stages, namely, child-supporting and old-age households, child-supporting households, old-age households, and unburdened households, using the ages of 16 and 65 as the thresholds (44).

### 3.4 Agricultural socialization services variable

The level of adoption of agricultural socialization services by farmers is closely related to the degree of local agricultural mechanization. Then we use the rate of comprehensive agricultural mechanization in the village as a proxy (45). The comprehensive agricultural mechanization rate refers to the weighted sum of the three operation levels of machine plowing, machine sowing, and machine harvesting in crop production operations, and this indicator can reflect the development process of agricultural mechanization more scientifically.

### 3.5 Control variables

Referring to the existing research results (20, 23, 46), we mainly include the following control variables in the empirical analysis. In agricultural production and operation, the head of the household often acts as the principal of the family to make decisions about the production and operation of the household. Therefore, this paper chooses gender, age, and political identity as the control variables of the personal characteristics of the head of the household. Among them, political identity refers to whether the head of the household is a member of the Communist Party of China (CPC). Household situation is also an important factor influencing specialization, and we chose the cultivated land fragmentation degree, economic status, and village cadre as control variables at the level of household characteristics. In addition, we also considered the influence of village status on farmers’ production and operation by selecting a poor village, the village’s road to the county center, broadband, and the distance to the office as control variables at the level of village characteristics.

### 3.6 Estimation approach

We evaluated the effects of human capital on agricultural specialization with an econometric model of the following type:


$$Y_{i}=\alpha_{0}+\alpha_{1}R_{i}+\sum \delta_{j}Z_{\mathrm{ij}}+\varepsilon_{i}$$ (2)

In Equation 2, *Y*_i_ refers to agricultural specialization; *R*_i_ denotes the human capital of the farm household; $\alpha_{1}$ is our important estimated coefficient; *Z*_ij_ represents a series of control variables; and $\varepsilon_{i}$ is a random disturbance term. When measuring the level of human capital, to ensure that the elements are comparable, we have to dimensionless the indicators of different natures and units. Since all indicators are positive, we treat them with the Equation 3:


$$y_{\mathrm{ij}}=\frac{x_{\mathrm{ij}}-\min(x_{\mathrm{ij}})}{\max(x_{\mathrm{ij}})-\min(x_{\mathrm{ij}})}*0.99+0.01$$ (3)

Where x_ij_ denotes the value of the jth indicator for the ith farmer before standardization, y_ij_ indicates the value of the jth indicator for the ith farmer after standardization. Further, we determine the indicator weights *λ*_ij_ by the entropy weighting method and use the linear weighting method to find the human capital level *R*_i_ (Equation 4) of the farmers:


$$R_{i}=\sum {Y_{\mathrm{ij}}}^{*}\lambda_{\mathrm{ij}},\sum \lambda_{\mathrm{ij}}=1$$ (4)

In the mechanism test section, we use the mediation effect test stepwise regression method (Equations 5–7) to verify the role of the degree of development of agricultural socialization services in the impact of human capital on agricultural specialization:


$$Y_{i}=\alpha_{0}+\alpha_{1}R_{i}+\sum \delta_{j}Z_{\mathrm{ij}}+\varepsilon_{i}$$ (5)

$$M_{i}=\beta_{0}+\alpha_{2}R_{i}+\sum {\gamma_{j}}^{*}Z_{\mathrm{ij}}+\mu_{i}$$ (6)

$$Y_{i}=\alpha_{0}^{\prime }+\alpha_{3}R_{i}+\alpha_{4}M_{i}+\sum \theta_{j}Z_{\mathrm{ij}}+\omega_{i}$$ (7)

Where *M*_i_ is the variable of the agricultural socialization services, it’s the mediating variable. $\beta_{0},\alpha_{0}^{\prime }$、$\alpha_{2},\alpha_{3},\alpha_{4},\gamma_{j},\theta_{j}$ are the parameters to be estimated; $\mu_{i},\omega_{i}$ denote the random perturbation terms; and the rest of the variables and symbols are kept in the same way as above.

Since the human capital of farmers affects production and business decisions, and agricultural specialization also helps to accumulate knowledge of agricultural production and improve human capital. Therefore, there is an endogeneity problem in the baseline regression model. To solve this problem, we choose instrumental variables related to human capital for regression analysis and set up the following 2SLS model (Equations 8–9):


$$R_{l}=\rho_{0}+\rho_{1}{\mathrm{IV}}_{i}+\rho_{2}+\sigma_{1i}$$ (8)

$$Y_{i}=\gamma_{0}+\gamma_{1}R_{l}+\gamma_{2}Z_{\mathrm{ij}}+\sigma_{2i}$$ (9)

Peer effect theory suggests that an individual’s behavior is influenced by the behavior of others (47, 48). The level of human capital at the community level is strongly correlated with the human capital traits of farmers, but this value is less likely to affect individual decision-making. Using the average level of human capital at the village level as an instrumental variable helps to address the problem of selection bias and provides a firmer empirical basis for the analysis. Thus, we use the average human capital of the village (the average level of human capital of the sample farmers in the village other than the farmer) as an instrumental variable for the human capital of the farmers. Theoretically, farmers with high levels of human capital in the same village may share information on agricultural production with farmers with low levels to help them improve their agricultural productivity. However, whether or not a farm household adopts a specialized production structure is a joint decision on the division of labor within the household and is not influenced by the human capital of external farmers, possessing exogenous characteristics.

## 4 Results and discussion

### 4.1 Descriptive statistics

We provide summary statistics of the data in Table 1. Overall, these research samples had low levels of human capital, with an average human capital index of 0.450, an average index of 0.677 for agricultural specialization. There is still much room for improvement in agricultural socialization services, and the comprehensive mechanization rate is only about 44.2%. In the sample, more than 90% of the heads of households were male, with an average age of about 56, and less than 40% were members of the CPC. In terms of household characteristics, the average number of plots is about 6, and about 7% of households have members who are village officials. In terms of village characteristics, about 68% of villages are not poor villages, 87% of villages are covered by broadband, village committees are about 7 km away from the township government or street office, and more than 98% of villages have concrete or slab-oiled roads leading to the county town center.

**Table 1:** Descriptive statistics.

Variable	Description	Mean	SD
Agricultural specialization	Herfindahl–Hirschman Index (HHI)	0.677	0.264
Human capital	Human capital index values measured using the entropy weight method	0.450	0.157
Gender	The gender of the head of household: 0 = Female; 1 = Male	0.917	0.275
Age	The age of the head of household	56.001	11.319
Political identity	Whether the head of household is a member of the Communist Party of China (CPC): 0 = No; 1 = Yes	0.398	0.490
Cultivated land fragmentation degree	Number of plots	5.946	6.284
Economic status	General trends in the situation of families over the past 2 years	2.967	1.145
Village cadre	Whether someone in the household is a village cadre: 0 = No; 1 = Yes	0.067	0.249
Poor village	Whether it is a poor village: 0 = No; 1 = Yes	0.316	0.465
Village’s road to the county center	1 = Dirt road; 2 = Cement road; 3 = Suet	2.459	0.524
Broadband	Whether the village is covered by broadband: 0 = No; 1 = Yes	0.866	0.341
Distance to office	The distance of the village committee from the township government or street office: Kilometers	7.241	6.747
Agricultural socialization services	The rate of comprehensive agricultural mechanization in a farmer’s village	0.442	0.415

### 4.2 Regression results

#### 4.2.1 Association between human capital and agricultural specialization

Table 2 shows the baseline regression results of the impact of human capital on agricultural specialized management. Where model (1) is the regression results when no control variables are added, model (2) to (4) are the regression results when head of household characteristics, household characteristics and village characteristics variables are added gradually. We find that human capital consistently affects agricultural specialization significantly and positively at the 1% statistical level in the process of adding no control variables and gradually adding control variables, in other words, as the level of human capital increases, farmers are more inclined to adjust their cropping structure toward specialization. Compared to low human capital farmers, better human capital means higher economic productivity (49); they have better access to information and acceptance of new things and ideas, and are more willing to adopt specialized production operations.

**Table 2:** Impact of human capital on agricultural specialization.

Variables	Model (1)	Model (2)	Model (3)	Model (4)
Human capital	0.075***(0.021)	0.071***(0.021)	0.078***(0.021)	0.080***(0.021)
Gender		−0.007 (0.012)	−0.002 (0.012)	−0.005 (0.012)
Age		−0.002***(0.000)	−0.002***(0.000)	−0.002***(0.000)
Political identity		−0.013*(0.007)	−0.013*(0.007)	−0.012*(0.007)
Cultivated land fragmentation degree			−0.004***(0.001)	−0.004***(0.001)
Economic status			0.011***(0.003)	0.011***(0.003)
Village cadre			−0.009 (0.013)	−0.010 (0.014)
Poor village				0.021***(0.007)
Village’s road to the county center				−0.035***(0.006)
Broadband				−0.011 (0.001)
Distance to office				−0.001*(0.001)
Constant	0.644***(0.010)	0.753***(0.023)	0.740***(0.026)	0.838***(0.031)
*N*	6,275	6,275	6,275	6,275
*R* ^2^	0.002	0.008	0.021	0.028
*F*	12.49	12.61	17.55	14.83

Robust standard errors are in parentheses; ***p < 0.01, *p < 0.1.

Further, based on the control variables, age, CPC, cultivated land fragmentation degree, village’s road to the county center, and the distance to the office have a significant negative effect on agricultural specialization. Older heads of households have fewer sources of off-farm income, are more dependent on agricultural land, prefer to grow subsistence crops such as wheat and rice for their survival needs, and tend to diversify their agricultural production. Households headed by CPC members tend to diversify. Possible reasons for this are that party members are more capable and have wider access to information, and they are more inclined to diversify their production to maximize income from agricultural operations. The large number of plots is not conducive to the participation of large-scale agricultural machinery in production activities, and small-scale farmers who are intensively cultivating their land are more likely to adopt diversified cropping structures based on the size of their plots and the topography of their land. The better the quality of the road to the county center, the easier it is for farmers to get to the county center to sell their produce, and the more they are willing to diversify their operations to maximize profits. Areas where village committees are distant from township or street offices may be less economically developed, with agricultural operations as their main source of income and less risk-resistant, and they have increased their economic resilience by diversifying their operations. Economic status and the village being a poor village have a positive effect on the degree of agricultural specialization. The high degree of specialization of households whose general household situation has deteriorated in the last 2 years may be explained by the fact that the deterioration of the general household situation makes it difficult for farmers to devote more energy to agricultural production and management, and that they can only reduce their agricultural inputs through specialization. Farmers in poor villages are more specialized than those in non-poor villages, possibly because poor villages have organizations such as village task forces to plan and train for the development of industries in the village and the production and management of villagers. There is no significant effect of gender, whether someone in the household is a village cadre, and whether the village is covered by broadband on agricultural specialization.

#### 4.2.2 Robustness and endogeneity discussion

Although more control variables have been included in the benchmark regressions in Table 2, there may still be potential factors that could bias the regression results and require further robustness checks. First, replace the explanatory variables. The fewer types of crops grown, the higher the specialization. We use the proxy variable method to characterize the agricultural specialization in terms of the concentration of the number of species (1/number of crop species grown) to test the robustness of the model. Second, the estimation model is replaced. We further divided the indicators of agricultural specialization into three categories of low, medium, and high levels of specialization, which were analyzed using the logit model. Third, subsample regressions. It has been found that older heads of households have declining human capital and lower human capital weakens specialization, while young heads of households have gradually accumulated human capital and agriculture is used more as a remedial strategy after job loss, and thus do not focus on specialization. Therefore, taking into account the reality of China’s agricultural production, we deleted the samples whose heads of household were under 45 years old and over 65 years old, and used subsample regression to test the robustness of the model.

On the basis of models (5) to (7) in Table 3, it can be found that after replacing the explanatory variables, replacing the estimation model and subsample regressions for the study sample in turn, human capital has a crucial positive effect on agricultural specialization, which is in line with the basic conclusions of the previous section, and the results are relatively robust.

**Table 3:** Robustness test.

Variables	Model (5)	Model (6)	Model (7)
Human capital	0.095***(0.024)	0.635***(0.162)	0.092***(0.026)
Control variables	Yes	Yes	Yes
Number of observations	6,275	6,275	4,017
*R* ^2^	0.040		0.026
*F*	19.04		9.44
Wald chi^2^		133.70	
Pseudo *R*^2^		0.014	

Robust standard errors are in parentheses; ***p < 0.01.

Further, Table 4 shows the regression results of using the instrumental variable method to solve the endogeneity problem. The coefficients of the core explanatory variables of the model are positive and highly significant, consistent with the results of the previous study, and the *p*-values of the instrumental variables Kleibergen-Paap rk LM are all zero, and the Cragg-Donald statistic is greater than the critical value at the 10% level of the weak identification test. The *F*-statistic for the first stage is greater than 10, indicating that there is no weak instrumental variable problem. These prove that the instrumental variables that we have selected are reasonable. This implies that after using the instrumental variables approach to deal with endogeneity, human capital still contributes significantly to agricultural specialization.

**Table 4:** Endogeneity test.

Variables	Model (8)	Model (9)
	First stage	Second stage
Human capital		1.001***(0.144)
IV	0.439***(0.032)	
Control variables	Yes	Yes
Constant	0.303***(0.022)	0.376***(0.080)
*N*	6,275	6,275
*F*	29.63	

Robust standard errors are in parentheses; ***p < 0.01.

#### 4.2.3 Impact pathways

Compared with traditional agriculture, modern agriculture pays more attention to profit and efficiency, and agricultural products produced in a specialized and industrialized mode are sold at a higher return, which makes the marginal output value of farmland specialized in agriculture higher (50). The results of the test in Table 5 show that there is a significant positive impact of human capital on the degree of agricultural socialization services and a significant positive effect of the agricultural socialization services on specialization. Incorporating both into the same model, there is still a significant positive effect of human capital and agricultural socialization services on agricultural specialization. From the methodology of the significance test of the mediation effect, it is clear that agricultural socialization services plays a partial mediating role in the impact of human capital on agricultural specialization. Possible reasons for this are that the higher the level of human capital and the stronger the technical skills of farmers in employment, the higher the rate of return to their labor, while the use of agricultural machinery can replace labor for large-scale production, the better the agricultural socialization services, the stronger the rate of labor substitution, and the more time that farmers can devote to non-agricultural employment to raise their incomes, and thus the degree of specialization in agriculture is higher.

**Table 5:** Mechanism analysis.

Variables	Agricultural socialization services	Agricultural specialization	
Human capital	0.077** (0.032)		0.079*** (0.021)
Agricultural socialization services		0.021*** (0.008)	0.020** (0.008)
Control variables	Yes	Yes	Yes
Constant	0.213*** (0.048)	0.872*** (0.030)	0.833*** (0.031)
*N*	6,275	6,275	6,275
*R* ^2^	0.069	0.027	0.029
*F*	53.17	14.35	14.36

Robust standard errors are in parentheses; ***p < 0.01, **p < 0.05.

#### 4.2.4 Heterogeneous results

We test for heterogeneous effects in terms of different regional types, different arable land operation sizes, the presence of land transfers, and the level of social networks.

Table 6 gives the results of the heterogeneity regression for region type. From the region, firstly, the human capital of farmers in the major grain-producing regions (MGPRs) and non-grain-producing regions (NGPRs) positively affects agricultural specialization at the statistical level of 1 and 10%, respectively. Secondly, from the point of view of economic development, the effect of human capital of farmers on agricultural specialization in the eastern region is not significant, while human capital in the central and western regions positively affects agricultural specialization at the 10% statistical level. This suggests that there is regional heterogeneity in the effect of human capital on agricultural specialization.

**Table 6:** Heterogeneity analysis of different regional types.

Variables	Type of production area		Level of economic development		
	MGPRs	NGPRs	Eastern	Central	Western
Human capital	0.079***(0.028)	0.057*(0.032)	0.010(0.041)	0.067*(0.030)	0.084*(0.039)
Control variables	Yes	Yes	Yes	Yes	Yes
*N*	3,618	2,657	1,630	2,906	1739
*R* ^2^	0.038	0.063	0.040	0.021	0.036
*F*	13.07	13.76	6.38	5.76	5.68

Robust standard errors are in parentheses; ***p < 0.01, *p < 0.01.

Table 7 gives the results of the heterogeneity regressions of business scale and land turnover. From the perspective of business scale, many scholars at home and abroad have not yet reached a consensus. We follow the World Bank’s definition of small farmers, which defines those whose family operates a land area of less than 30 mu as small farmers, and those whose family operates a land area of more than or equal to 30 mu as large-scale farmers. As can be seen from Table 7, human capital significantly increases the degree of specialization of small-scale farmers at the 1% statistical level and does not have a significant effect on large-scale farmers. Possible reasons for this are that large-scale households are more dependent on farm income, and that specialization comes with greater risks and rewards, and that some farmers tend to adopt specialization to improve their farm income, while others tend to diversify their farming to reduce the risks of farming operations, and thus do not focus on specialization. The difference is that smallholder farmers operate on a smaller area and may obtain similar returns from specialized and diversified production. The adoption of diversification, on the other hand, requires the procurement of a greater variety and smaller quantities of production materials, which tends to create diseconomies of scale. Therefore, smallholders prefer specialization to reduce transaction costs and increase comparative returns from cultivation.

**Table 7:** Heterogeneity analysis of different scales and land transfer.

Variables	Small farmers	Large-scale farmers	Land transfer	Non-land transfer
Human capital	0.080***(0.022)	−0.019(0.065)	0.065(0.043)	0.085***(0.024)
Control variables	Yes	Yes	Yes	Yes
*N*	5,776	499	1,600	4,675
*R* ^2^	0.030	0.048	0.034	0.029
*F*	13.47	2.39	4.62	11.40

Robust standard errors are in parentheses; ***p < 0.01.

Concerning land transfer, human capital significantly increased the specialization of farmers without land transfer at the 1% statistical level and had a non-significant effect on farmers with land transfer. Possible reasons for this are that the production patterns of farmers without land transfers are more stable than those with land transfers, and specialization helps them to build up their skills and increase their income from agricultural operations. As can be seen, the promotion of the land transfer policy has brought about great changes in rural production and life (51).

Social networks are relatively stable relationships formed by interactions between individual members of a society. In this paper, the sum of farmers’ expenses, such as communication and internet, is used as a proxy indicator of the social network situation. Meanwhile, this study categorized the farmers into low, medium, and high levels of regression estimation according to the level of social network as shown in Table 8. The results show that the effect of human capital on specialization is not significant for farmers with a low social network rank. The effect of human capital on specialization is significant at the 10 and 1% statistical levels, with positive coefficients for farmers with medium and high social network class, respectively. The possible reason for this is that social networks help to improve farmers’ access to information. When farmers’ social networks are high, they may communicate more closely with new agricultural business entities, agricultural machinery service organizations, and large growers, and will take the initiative to imitate and learn from their business models. And these business entities usually adopt a large-scale, specialized approach to agricultural production to save transaction costs. At the same time, the social network is a key channel for farmers to obtain information on agricultural production, and it is a complementary means to the differences in the ability to obtain information caused by the educational differentiation of farmers, which is conducive to promoting the improvement of their production mode and production efficiency.

**Table 8:** Heterogeneity analysis of the level of social networks.

Variables	Low	Medium	High
Human capital	0.035(0.032)	0.075*(0.040)	0.159***(0.041)
Control variables	Yes	Yes	Yes
*N*	3,203	1,598	1,474
*R* ^2^	0.037	0.036	0.034
*F*	9.59	5.14	4.94

Robust standard errors are in parentheses; ***p < 0.01, *p < 0.01.

### 4.3 Discussion

From a food security perspective, human capital and agricultural socialization services have important implications for specialization. The former can promote specialized production by upgrading the quality of farmers, while the latter can integrate resources and provide professional services to help agricultural production. Highly qualified workers have a high level of scientific literacy and are well versed in modern agricultural production and management. They are more willing to try and promote new technologies and varieties, and to promote the transformation of food production into intensification, industrialization and specialization. Agricultural socialized service organizations are able to centralize the procurement of production materials and unify mechanical operations by aggregating the needs of farmers, and use information technology such as big data and cloud computing to empower production and operation. This reduces the physical costs of growing food, improves productivity and provides technical support for food security.

This study found that agricultural socialization services play an important mediating role before human capital and agricultural specialization. Human capital is a fundamental element in the production and operation of farming households, especially in the context of dramatic demographic changes. Currently, China’s rural population is aging. At the same time, the young and strong labor force in the countryside continues to move to the non-agricultural fields in the cities, and agricultural production will face a shortage of labor (52). The rise of agricultural social services has, to a certain extent, compensated for the shortage of rural labor in terms of quantity and quality and increased labor productivity (48). At the same time, the services provided by agricultural service organizations are premised on a certain scale, and agricultural machinery has a dedicated type, which makes it easy to induce farmers to form a pattern of continuous cultivation. In addition, we find that the contribution of human capital to specialization is stronger for farmers with high levels of social networks. This may be since social networks help to improve farmers’ access to information and financial support (53). When farmers’ social networks are high, they may communicate more closely with new agricultural business entities, agricultural machinery service organizations, and large growers, and will take the initiative to imitate and learn from their business models. And these business entities usually adopt a large-scale, specialized approach to agricultural production to save transaction costs. At the same time, the social network is a key channel for farmers to obtain information on agricultural production, and it is a complementary means to the differences in the ability to obtain information caused by the educational differentiation of farmers, which is conducive to promoting the improvement of their production mode and production efficiency.

It is worth noting that our study has some limitations. In the future, we may explore this in a variety of ways. Qualitative research, for example, can contribute to a deeper understanding of the issue. In the future, we intend to conduct in-depth interviews with farmers through field research. At the same time, it maintains close contact with farmers and seeks to be able to obtain tracking data for three to five complete production cycles for further case studies. This study utilizes cross-sectional data that do not capture the dynamics of human capital. Although this paper discusses the role of agricultural socialization services in promoting specialization, it does not analyze in depth the number of adopted links of agricultural socialization services, expenditure on service fees, service modes, contractual status, and other indicators of individual farmers. In the future, we can obtain more specific and detailed data related to agricultural socialization services through field research and expand it into panel data, taking into account the time dimension to test the long-term dynamic effects.

## 5 Conclusion and policy implications

Agriculture is the basis of the national economy, and the division of labor is the source of economic growth. As agricultural transformation is closely linked to the level of division of labor, specialization is a key indicator of upgrading the horizontal division of labor. Because of this, the government of China has implemented various interventions to improve labor productivity, such as agricultural training, productive agricultural services, and agricultural support and protection subsidy policies. The objective of this study is to analyze the impact of human capital on agricultural specialization. The study uses large-scale micro-research data representative of China’s rural production and operation, and applies the entropy method to measure the comprehensive human capital level of farm households, using the instrumental variables method and a variety of robustness tests to address many potentially relevant sources of endogeneity, as well as providing knowledge enhancement for research in the field of the division of labor economy, and compensating for the lack of research on a single human capital factor in the field of agriculture.

The results show that the level of human capital in China is generally low and the degree of specialization is not high. Human capital has a significant positive effect on agricultural specialization. These findings suggest that there is still much room for improvement in the level of human capital in China, and that the improvement of human capital can change the way of agricultural production and management and accelerate the process of agricultural modernization.

Further mechanism analyses showed that improvements in human capital increased the level of agricultural socialization services and that the increase in the level of agricultural socialization services was positively correlated with the results of agricultural specialization. This suggests that the impact of human capital on agricultural specialization is not only driven by objective adjustments in production decisions, but also relies on pathways based on agricultural socialization services. This further enriches the theory of the division of labor in agriculture.

Heterogeneity analysis further shows that the role of human capital level is more pronounced in the main food-producing areas, non-main producing areas, central and western regions, with a significant contribution to smallholders and farmers without land transfers. This suggests that there are differences in the role of human capital in different contexts, and that it is important to tailor the approach to the local context.

Overall, first, strengthening investment in rural human capital. Improvement of the rural health care system and enhancement of the level of rural human capital through the construction of village health clinics and free medical check-ups. Through vocational education and training, we will improve the digital literacy of farmers, train new farmers who can operate modern agricultural machinery and master modern agricultural technology, and inject new vitality into the modernization of agriculture and rural areas. Secondly, play the role of financial funds to guide, anchor agricultural machinery and equipment intelligence and other cutting-edge technologies, relying on research institutes to carry out research. Support the development of leading enterprises in the agricultural machinery and equipment industry, and encourage cross-industry mergers and acquisitions. It will incorporate appropriate mechanization into the conditions for the construction of high-standard farmland, create an agricultural machinery research and development group with regional suitability, and attack weak links such as grain drying, seed breeding, and precision fertilizer and medicine application. For example, developing small agricultural machinery that is more suitable for hilly and mountainous areas can avoid difficulties in operation due to small plots and steep slopes. It is also possible to develop composite agricultural machinery that integrates functions such as sowing, fertilizing, weeding, and harvesting, thereby reducing the hassle and cost of farmers having to replace equipment and lowering the technical barriers to using agricultural machinery. At the same time, it is necessary to strengthen the construction of agricultural cooperatives, professional and technical associations and other types of socialized organizations, and to strengthen the ability of agricultural service organizations to link up with and bring farmers. Thirdly, deepening the reform of the land system and promoting the transfer of land and moderate-scale operations. Improve the land transfer contract mechanism to stabilize the expectations of business entities.

## Data Availability

The datasets presented in this study can be found in online repositories. The names of the repository/repositories and accession number(s) can be found: https://ssec.zju.edu.cn/2020/0424/c86173a3031114/page.htm.

## Footnotes

^1^ http://200.200.201.247/rwskdataindex.htm
